# The Prevalence of Diabetic Retinopathy in the 21st Century: New Insights From a Portuguese Center

**DOI:** 10.7759/cureus.85501

**Published:** 2025-06-07

**Authors:** Pedro Moreira Martins, Sara Leite, Sofia Teixeira, João Castro Cabanas, Luís Silva, Filipe Sousa-Neves

**Affiliations:** 1 Ophthalmology, Unidade Local de Saúde de Gaia e Espinho, Porto, PRT; 2 Cardiovascular Research and Development Center - UnIC@RISE, Unidade Local de Saúde de Anta, Porto, PRT

**Keywords:** diabetic retinopathy, diabetic retinopathy detection, diabetic retinopathy management, diabetic retinopathy prevalence risk factors screening strategies therapeutic interventions diabetes mellitus ocular complications visual impairment, type-ii diabetes mellitus

## Abstract

Introduction

Diabetic retinopathy (DR) is a leading cause of vision loss among working-age adults; yet, robust prevalence data remain scarce. In this study, we aimed to determine the prevalence of DR and identify its main associated risk factors in adults with type 2 diabetes mellitus (T2DM) monitored at a family health unit in northern Portugal

Methods

This retrospective study evaluated all patients at a family health unit diagnosed with T2DM between 2002 and 2013. Electronic records were screened for retinal evaluations, *via* the national screening or opportunistic exams. All patients who had undergone a retinal exam 10 years after their diabetes mellitus (DM) diagnosis were included. Baseline data - including gender, age, diabetes duration, glycated hemoglobin, smoking history, and hypertension - were collected, along with DR presence and its classification. The main outcomes were prevalence, type, risk factors, and complications of DR.

Results

Between 2002 and 2013, 355 patients were diagnosed with T2DM at this family health unit. Of these, 64 were excluded due to the absence of a retinal evaluation at the 10-year follow-up. Among the 294 included patients, 152 were female, with a mean age at diagnosis of 71.1 ±12.1 years. Most (257/294) were evaluated through the national screening program, with the remaining being evaluated through an opportunistic appointment. Overall, 18 patients were diagnosed with diabetic retinopathy (point prevalence: 6.12%). Of these, 14 were male (78%), four developed macular oedema, eight underwent LASER treatment, and one required *pars plana* vitrectomy for proliferative DR. Patients with DR had worse glycemic control (mean glycated hemoglobin: 7.67 ±1.07 vs. 6.61 ±0.74 in those without retinopathy, *p*<0.001) and a higher need for insulin (33% vs. 8.3%, *p*=0.002). Hypertension and smoking habits did not significantly differ between groups.

Conclusions

DR prevalence and risk factors vary in the literature due to differences in studied populations and methodologies. Reported prevalence ranges from 20 to 35% over 5-10 years and is closely associated with poor glycemic control and the need for insulin therapy. The significantly lower rate observed in our study may reflect earlier diagnosis and improved access to treatment, highlighting the importance of systematic screening and proper glycemic control in patients with T2DM. Early identification of these patients can provide tailored follow-up strategies, guide timely therapeutic interventions, and ultimately help prevent vision-threatening complications. Furthermore, our results may serve as a foundation for further research on the current impact of T2DM on ophthalmic health services. Larger, multicentric studies are needed to provide a more comprehensive and up-to-date picture of DR within today’s healthcare systems.

## Introduction

Diabetes mellitus (DM) is a chronic metabolic disorder characterized by elevated blood glucose levels resulting from defects in insulin secretion, insulin action, or both. It leads to significant morbidity and mortality worldwide, mainly related to the micro- and macrovascular complications, which include myocardial infarction, stroke, renal failure, and diabetic retinopathy (DR) [1]. Although several subcategories of the disease have been described, two main types account for the vast majority of affected patients: type 1 and type 2. Type 1 DM is an autoimmune disorder in which the body's immune system targets the insulin-producing beta cells in the pancreas, leading to an absolute deficiency of insulin. It typically manifests in children and young adults. In contrast, type 2 DM (T2DM), which accounts for the majority of diabetes cases, is characterized by insulin resistance and a relative deficiency in insulin secretion. It commonly occurs in adults over the age of 45 but is increasingly being diagnosed in younger populations, including children and adolescents, due to rising obesity rates [2]. The International Diabetes Federation estimates that more than 500 million people have diabetes, a number that is on the rise and is predicted to reach 780 million by 2045. Of these, nearly seven million are expected to die from diabetes-related complications, which places a significant burden on healthcare costs and the workforce [3]. In Portugal, approximately one million individuals are diagnosed with DM, with an additional 400,000 estimated to have undiagnosed DM, resulting in a prevalence of 9.1% [3].

DR is a microvasculopathy and one of the most common complications of DM, and the leading cause of vision loss in working-age adults worldwide [4]. Despite its significance as a major healthcare challenge of the 21st century, the literature regarding the prevalence and risk factors of DR remains limited and highly heterogeneous, primarily due to variations in studied populations and research methodologies. A recent systematic review and meta-analysis estimated the global prevalence of DR to be 22.3% [5]; however, this figure may not be directly applicable to the Portuguese population, as genetic factors and the nuances of the healthcare system can influence both the diagnosis and treatment of DM. As such, there is limited literature that accurately describes the baseline characteristics, prevalence, primary complications, and treatment for DR in Portugal. In this study, we aim to provide a comprehensive description of the overall prevalence of DR, associated risk factors, glucose control, and main complications, based on a detailed analysis of a cohort of patients with T2DM under surveillance at a primary healthcare unit in northern Portugal.

## Materials and methods

Study design

This was a retrospective cohort study conducted at a single family health unit in the district of Porto, within the influence area of the Gaia/Espinho Health Unit. The entire electronic health record database was reviewed, and all patients newly diagnosed with T2DM between 2002 and 2013 were included. These patients were identified based on coding by their Family Medicine physician.

This timeframe was selected because 2002 marks the first year with electronic records available from the national DR screening program. From that year onward, all patients diagnosed with T2DM and registered with a general practitioner have been systematically referred to a screening center equipped with a non-mydriatic fundus camera (Canon CR-2, Tokyo, Japan). Patients were excluded from the screening program if they had bilateral blindness, had attended at least one ophthalmology appointment in previous six months, or were already under follow-up at a retina consultation due to DR. Fundus imaging is performed using two 45º nonstereoscopic retinal photographs per eye, one centered on the optic disc and another on the posterior pole. These images are then uploaded to the Reading Centers, reviewed by trained ophthalmologists, and classified into the following categories, according to the guidelines of the Portuguese Directorate-General of Health (Table 1) [6].

**Table 1:** Classification of DR according to the guidelines of the Portuguese Directorate-General of Health DR: diabetic retinopathy; LASER: light amplification by stimulated emission of radiation

Classification of DR
R0: no diabetic retinopathy
R1: mild non-proliferative DR
R2: moderate non-proliferative DR
R3: severe non-proliferative DR or proliferative DR
M1: maculopathy
V1: high-risk proliferative DR or tractional retinal detachment
ICN: inconclusive
P0: individuals who underwent LASER treatment with stable DR
P1: individuals with DR in which LASER treatment is deemed insufficient

If only one eye was available, the DR was graded based on that eye only; if evaluation differed among eyes, the worse grade was used in the analysis. In cases of mild/moderate DR, the presence of maculopathy was considered the key characteristic and was used in the final classification.

Patient selection and data collection

This study used anonymized, retrospective data extracted from electronic health records. All patient information was anonymized before analysis to ensure confidentiality. 

Patients were included if they had undergone at least one ophthalmologic evaluation, either through the national DR screening program or an ophthalmology consultation, at the 10-year mark after the T2DM diagnosis. To ensure consistency in data interpretation and facilitate statistical analysis, DR classification from ophthalmology consultations was standardized according to the national DR screening system categories. The inclusion period for newly diagnosed T2DM patients was limited to 2013 to allow for a full 10-year follow-up. For eligible patients, the following data were extracted from the electronic health records: age, gender, glycated hemoglobin (HbA1c, recorded annually for 10 years), smoking status, presence of hypertension, need for insulin therapy, DR classification, complications, and respective treatment. Hypertension was defined as a coded diagnosis in the medical record during follow-up. Smoking status was categorized as current, former, or never, according to the most recent information in the medical records. Need for insulin therapy was determined by documentation of insulin use during the 10-year follow-up period in the medication records.

Interim evaluations and assessment of DR progression over time were not included, as the number of patients with complete interim data was insufficient for a statistically robust analysis. Missing data were handled using complete case analysis; only patients with available data for the variables required in each specific analysis were included. For example, if HbA1c was missing for a patient in a given year, that year’s value was excluded from longitudinal analysis, but the patient was still included for other available years/variables. No data imputation was performed. Data were checked for consistency and obvious errors (e.g., implausible values, duplicates) before analysis, and any such records were excluded.

Statistical analysis

Statistical analysis was conducted using SPSS Statistics V 30 (IBM Corp., Armonk, NY). Descriptive statistics were used to summarize patient characteristics, with continuous variables presented as mean ± standard deviation (SD) and categorical variables as absolute frequencies and percentages. Comparative analyses between groups were conducted using t-tests or Mann-Whitney U tests for continuous variables and chi-square tests for categorical variables. A p-value <0.05 was considered statistically significant. To identify independent risk factors for DR at the 10-year follow-up, a multivariate logistic regression analysis was performed, including age, sex, hypertension, smoking status, insulin use, and mean HbA1c as covariates. Additionally, a model-based sensitivity analysis was conducted for the subgroup of patients who did not have a 10-year ophthalmologic evaluation. Specifically, logistic regression coefficients obtained from the evaluated patients were applied to the excluded patients with complete predictor data to estimate their individual probabilities of having DR, and the sum of these probabilities was used to adjust the overall prevalence for the entire cohort.

## Results

A total of 355 patients were diagnosed with T2DM between 2002 and 2013 in the analyzed family health unit, where the prevalence of T2DM stood at 9.51% as of February 2025. Of these, 64 patients lacked an ophthalmologic evaluation at the 10-year follow-up and were therefore excluded from the study. The final cohort comprised 294 patients, of whom 152 (51.7%) were female. The mean age at the time of diabetes diagnosis was 71.1 ±12.1 years.

Among the 294 included patients, 257 (87.4%) were evaluated through the national electronic DR screening program. A total of 514 retinal assessments were evaluated at the 10-year mark, with the following classifications: 244 patients were classified as R0 - no DR; six patients were classified as R1 - mild non-proliferative DR; two patients were classified as R2 - moderate non-proliferative DR; two patients were classified as M1 - maculopathy; two patients were classified as P0 - patients that underwent laser therapy with stable DR; and one assessment was inconclusive (ICN), due to poor image quality from advanced cataract (the retina appointment after cataract surgery revealed bilateral mild non-proliferative DR - R1).

In addition to the national DR screening program, 37 diabetic patients underwent an ophthalmology appointment (either referenced from the electronic screening program or due to other clinical motives). Twenty-five did not show any signs of DR at the 10-year mark. Of the remaining 12 patients, 10 were referred from the DR screening (four due to maculopathy - M1, two due to moderate non-proliferative DR - R2, and four due to poor image quality from an advanced cataract). At the end of the follow-up period, the majority (10 patients) were classified as M1, with three having mild non-proliferative DR and seven having moderate non-proliferative DR; one patient had mild non-proliferative DR (R1), and another showed signs of proliferative DR (R3). The DR classification of the included patients is presented in Table 2.

**Table 2:** DR classification of the 294 type II diabetic patients included in the study R0: no DR; R1: mild non-proliferative DR; R2: moderate non-proliferative DR; R3: proliferative DR; M1: maculopathy; P0: patients who underwent LASER therapy with stable DR DR: diabetic retinopathy; ICN: inconclusive; LASER: light amplification by stimulated emission of radiation

	R0	R1	R2	R3	M1	P0	ICN
Electronic screening (n=257)	244	6	2	-	2	2	1
Ophthalmology appointment (n=37)	25	1	-	1	10	-	-

After cross-checking the information gathered from both platforms, 18 out of the 294 evaluated diabetic patients were diagnosed with some degree of DR, corresponding to a point prevalence of 6.12% 10 years after the inaugural diagnosis of T2DM. Of these, 14/18 were male (78%), with a similar age at diagnosis when compared with non-DR patients (69.6 vs. 71.2 years, p=0.39).

**Figure 1:**
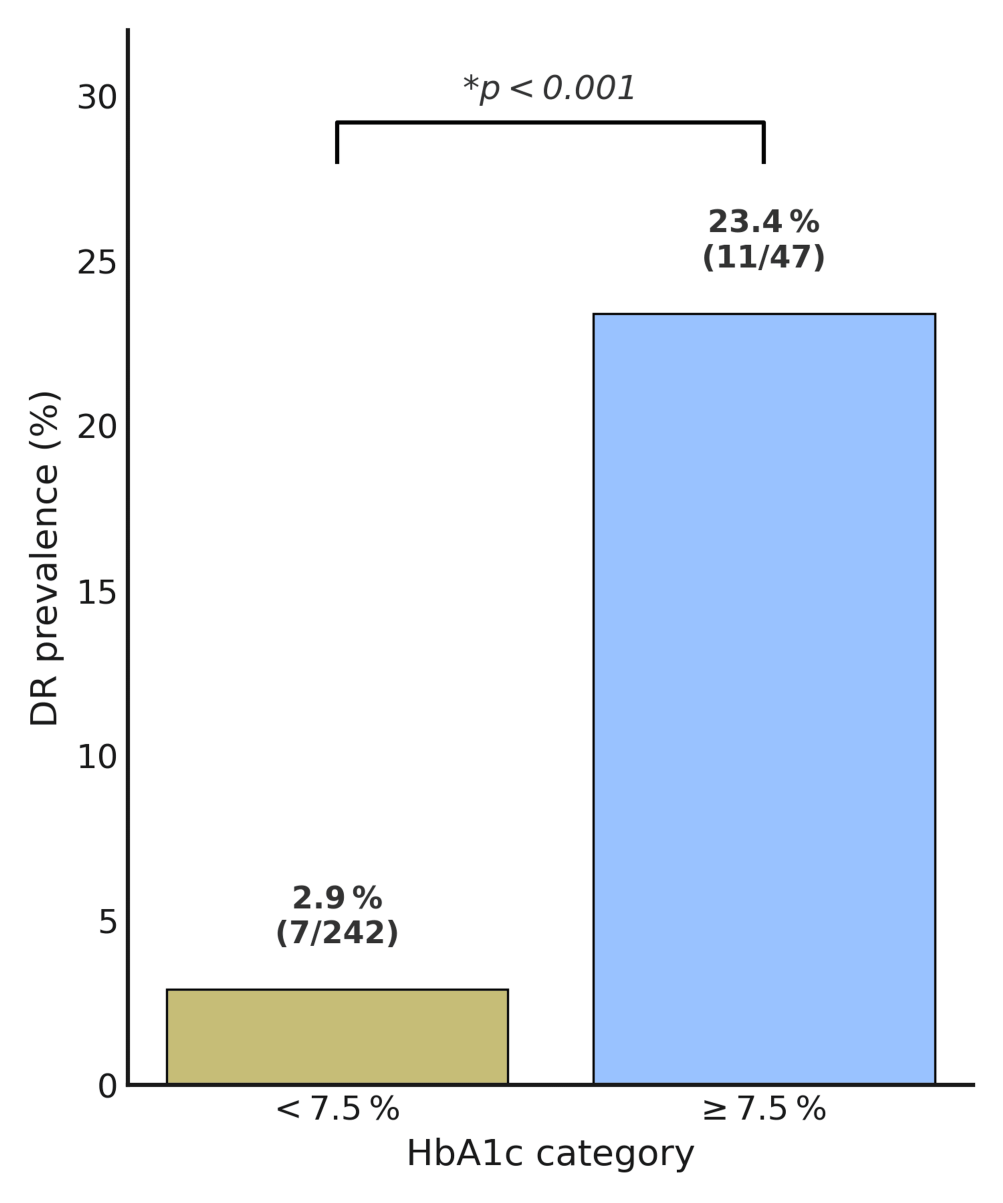
Prevalence of diabetic retinopathy 10 years after T2DM diagnosis, stratified by mean HbA1c DR: diabetic retinopathy; HbA1c: glycated hemoglobin; T2DM: type 2 diabetes mellitus

The comparison of baseline clinical characteristics and glucose control between T2DM patients with and without DR is shown in Table 3. Past and current smoking habits were more prevalent in DR patients (39%) compared to non-DR patients (18%), although the difference did not reach statistical significance (p=0.052). Hypertension was present in 78% of DR patients and 81% of non-DR patients (p=0.72). HbA1c levels over the 10 years were significantly higher in DR patients (7.67 ±1.07%) than in non-DR patients (6.61 ±0.74%, p<0.001). Notably, DR was present in only 2.8% of patients with HbA1c levels below 7.5%, compared to 23.4% in those with HbA1c ≥ 7.5%, a difference that was statistically significant (χ²=24.95, p<0.001) (Figure 1).

**Table 3:** Baseline characteristics, 10‑year glycemic control, and ocular complications/interventions in patients with T2DM, stratified by the presence of DR ^*^Statistically significant DR: diabetic retinopathy; HbA1c: glycated hemoglobin; LASER: light amplification by stimulated emission of radiation; SD: standard deviation; T2DM: type 2 diabetes mellitus

	DR	Non‑DR	Total	P‑value (DR vs. non-DR)
Number of patients	18	276	294	-
Point prevalence of DR, %	-	-	6.1%	-
Age at T2DM diagnosis, years, mean ±SD	69.6 ±9.1	71.2 ±10.2	-	0.39
Past + current smokers, n (%)	7 (38.9 %)	50 (18.1 %)	57 (19.4 %)	0.052
Hypertension, n (%)	14 (77.8 %)	224 (81.2 %)	238 (81 %)	0.72
Mean HbA1c over 10 years, %, mean ±SD	7.7 ±1.1	6.6 ±0.7	6.7 ±0.8	<0.001^*^
Insulin therapy, n (%)	6 (33.3 %)	23 (8.3 %)	29 (9.9 %)	0.002^*^

A total of 29 included patients were treated with insulin, with six of them having DR (20.7%). In line with this data, insulin therapy was significantly more frequent among DR patients, with 33% requiring insulin compared to only 8.3% in the non-DR group (p=0.002). In a multivariate logistic regression model, only mean HbA1c was significantly associated with DR at 10 years (OR=3.03; p<0.001). Hypertension (p=0.80), smoking status (p=0.30), and insulin use (p=0.28) were not significantly associated with the presence of DR.

Regarding DR complications and treatment, diabetic macular edema was present in four patients (three had moderate DR with maculopathy and one presented with proliferative DR); two were treated with intravitreal anti-vascular endothelial growth factor (VEGF) injections. A total of eight patients required focal or panretinal laser treatment, and one patient with proliferative DR underwent pars plana vitrectomy (Table 4).

**Table 4:** Ocular complications/interventions in the DR cohort at the 10-year follow-up DR: diabetic retinopathy; LASER: light amplification by stimulated emission of radiation; VEGF: vascular endothelial growth factor

Complication/treatment	N	% of DR cohort
Diabetic macular edema	4	22%
Focal or pan‑retinal LASER photocoagulation	8	44%
Intravitreal anti‑VEGF injections	2	11%
Pars plana vitrectomy	1	6%

Some data were available for the 64 excluded patients. Mean HbA1c, available for 12 of these patients, was 6.89% - a value not significantly different from the group of patients without DR (p=0.27). Also, 17 of the 64 excluded patients underwent some form of ocular evaluation during the 10-year follow-up period, although, as previously explained, none had an evaluation at the 10-year mark. Among them, only one patient had a positive screening for DR (M1) two years after the diagnosis of T2DM, but did not undergo further evaluation. The remaining patients had either no signs of diabetic retinopathy (14 classified as R0) or had an inconclusive screening result (two classified as ICN). To assess the potential impact of missing data on overall prevalence estimates, a model-based sensitivity analysis was conducted. The multivariate logistic regression model was applied to the 10 excluded patients with complete risk factor data, yielding an expected number of DR cases of approximately 0.85 (less than one case). Including these predictions, the adjusted prevalence of DR for the entire cohort was estimated at 6.2%, which is nearly identical to the observed prevalence among evaluated patients alone (6.12%).

## Discussion

DR remains a significant health hazard for individuals with DM, as it can lead to vision impairment and blindness if not properly managed. In fact, as the main cause of vision loss in working age adults, vision-threatening DR has been associated with disintegration of social interaction and social life, result in stress, tension and irritation among family members, and is often one of the main drivers of unemployment and loss of income in this age group [7]. It also poses a significant burden on medical personnel hours, and with the progressive shift towards treatment with intravitreal injections, the cost per treated DR patient has significantly risen in the past two decades: a recently published study in an hospital in Oslo estimates this metric has changed from €2,935 in 2010 to €3,665 in 2018 [8].

In Portugal, DM has a high prevalence. ​Of the one million individuals with DM aged 20-79 years old, over 40% are estimated to be undiagnosed. An additional one million people have impaired glucose tolerance; this also has an impact on mortality, with 22,858 DM related deaths in adults aged 60 years or less in 2021 [3]. As such, it is crucial to understand the epidemiological dynamics of DR, mainly the incidence, prevalence, and its associated risk factors, for implementing effective screening and preventive strategies, thereby reducing the risk of vision loss among these individuals. Identifying these factors enables healthcare professionals to target high-risk populations and optimize resource allocation to mitigate the impact of this sight-threatening condition [9]. The prevalence of DR varies across different populations and regions, influenced by factors such as genetic predisposition, healthcare infrastructure, diabetes management strategies, and socioeconomic conditions. However, despite being one of the greatest ophthalmological challenges of our era, the published data regarding prevalence and risk factors are scarce and highly heterogeneous.

Several landmark studies have investigated the prevalence of DR across different populations. The Wisconsin Epidemiologic Study of Diabetic Retinopathy (WESDR), initiated in 1979, is one of the largest and longest-running population-based studies on DR. It reported that after 20 years of DM, 99% of patients with type 1 and 60% of patients with type 2 exhibited some degree of retinopathy; in patients aged 30 years or older, with less than five years of disease duration, this prevalence drops to a still high value of 29% [10]. The United Kingdom Prospective Diabetes Study (UKPDS) surveyed 1,919 patients newly diagnosed with T2DM over six years. At the time of DM diagnosis, 703 (37%) exhibited signs of DR; 22% of patients without DR developed retinopathy within six years, and 29% of those with DR at baseline experienced progression of the disease [11].

The Liverpool Diabetic Eye Study (LDES) assessed DR prevalence in a UK urban population, with a 12.4/1,000 prevalence of DM and reported a rate of 45% in type II patients requiring insulin and 31.3% in type II patients who did not require insulin therapy [12]. ​The Diabetic Retinopathy Screening Service for Wales also conducted an analysis to determine the incidence of DR among individuals with T2DM who initially presented with no signs of retinopathy. Over four years, the cumulative incidence of any DR was 36.0% [13]. In the United States, the National Health and Nutrition Examination Survey (NHANES) showed a prevalence of any type of DR among adults aged 40 and older of 28.5% [14]. Finally, a recent systematic review and meta-analysis estimated the global prevalence of DR at 22.3%; furthermore, it estimated that in 2020 the number of adults worldwide with DR was 103 million, and that figure is projected to increase to 160 million by 2045 [5]. From the immense available data, prevalences vary significantly. It is difficult to compare studies and formulate appropriate conclusions, taking into account the high variability of the study protocols, especially when a significant proportion of these population-based studies were conducted decades ago and may not reflect current advancements in medical practices. As such, more recent and methodologically sound studies are needed to ascertain the clinical picture of DR, especially in our population.

The RETINODIAB study represents a pivotal effort in understanding the prevalence and progression of DR in the Portuguese population. This five-year retrospective study, carried out from July 2009 to October 2014, assessed the effectiveness of a community-based electronic screening program in the Lisbon and Tagus Valley regions for DR in individuals with T2DM. Two 45° nonstereoscopic retinal digital photographs were obtained per eye and further graded according to the degree of DR. Overall, 103,102 screening examinations were obtained. Women comprised 49.6% of all patients, and the mean duration of diabetes was 8.5 years. Any degree of DR was detected in 8,584 (16.3%) patients; of these, 10.4% had mild non-proliferative DR, 2.8% had moderate non-proliferative DR, 1.3% had severe non-proliferative DR, and 1.8% presented with proliferative DR [15]. A further analysis revealed that the annual incidence of DR among patients without retinopathy at baseline was 4.60% in the first year, decreasing to 3.87% by the fifth year [16]. These prevalence results are lower than most estimates from large-scale international studies.

Our retrospective cohort study, conducted at a family health unit in northern Portugal, analyzed a total of 294 patients diagnosed with T2DM between 2002 and 2013, trough fundoscopy or colour fundus photographs (from the national DR screening) to assess the presence of DR. After a 10-year follow-up, the point prevalence of any stage of DR was found to be 6.12%. This is considerably lower than the rates reported in both international and national studies. Several variables may contribute to the discrepant findings in our study, such as local public health policies and incentives that allow for earlier screening and diagnosis of DM, which certainly impact the detection and development of microvascular complications. Glycemic control is also a key player in the development of DR, and indeed, data from BI-CSP, a national platform that provides statistical information on primary healthcare in Portugal, shows that this family health unit demonstrated better control of T2DM patients compared to the national average. This may help explain some of the findings of this study and reinforces the importance of metabolic control in the prevention of DR. Consistently, the UKPDS study reported that a decrease of 1% of the HbA1c levels was associated with a 31% decrease in the risk of microvascular complications, including DR [11].

A recent study evaluating DR prevalence in adults with undiagnosed DM found asymmetric rates according to the values of HbA1c, varying from 6.9% for HbA1c <7.0% to 16.7% for HbA1c 9.0-9.9%, and 42.6% for HbA1c ≥10.0% [17]. In close association, a systematic review and meta-analysis also established the link between insulin therapy and increased risk of DR [18]. In our study, the mean HbA1c level over 10 years was significantly higher in patients with DR (7.67 ±1.07%) compared to those without DR (6.61 ±0.74) (p<0.001), with a much higher need for insulin therapy (33 vs. 8.3%, p=0.002), corroborating the impact of metabolic control in the development of DR. In fact, in a multivariate logistic regression model, the mean HbA1c was the sole predictor significantly associated with DR at 10 years (OR=3.03; p<0.001). Higher blood pressure has also been implicated as a risk factor for developing DR in many large-scale population-based studies, such as the UKPDS and the WESDR, although that association was not evident in our analysis, likely due to the high prevalence in both groups [10,11].

The relationship with the smoking status, however, is not as clear. While the reduced oxygen delivery and increased formation of advanced glycation end-products may contribute to higher rates of DR, the lower blood pressure associated with smoking, along with the unknown influence of some of the components in tobacco, might lead to a decreased risk of development of DR. A recent systematic review states that smoking is associated with an increased risk of DR in type I DM and reduced risk in T2DM, but raised important questions regarding the validity of the findings, manly related to how smoking-status is reported, and how the lower survival of smokers may lead to a shorter follow-up of these patients [19]. In our study, albeit non-significant, there was a trend towards a higher proportion of smokers in the DR group; more studies are needed to understand the impact of said variant on the incidence and progression of DR.

Limitations

This study has several limitations. It involves a retrospective, single-center analysis of a modest sample, limiting both statistical power and external validity. Additionally, 61 individuals (17% of the initial cohort) were excluded due to a lack of a 10-year ophthalmologic assessment. While a model-based sensitivity analysis suggested that the expected number of additional DR cases among these excluded patients was less than one, this estimate was based on only 10 individuals with complete risk factor data. As such, the adjusted prevalence (6.2%) should be interpreted with caution, although it is nearly identical to the observed prevalence among evaluated patients (6.12%). The available information does not reveal major differences in age or gender when comparing included and excluded patients, but the possibility of exclusion bias cannot be entirely ruled out. The use of mean HbA1c over 10 years as a single metric, while informative, does not accurately capture variability, time spent within target range, or patterns of glycemic deterioration, which could affect the incidence of DR.

As with all retrospective analyses, our findings depend on the accuracy and completeness of recorded data. Important potential confounders such as BMI, lipid profile, renal function, and medication adherence were not consistently available and therefore could not be included in the analysis. The omission of these variables limits our ability to fully control for residual confounding and may have influenced the observed associations, and, as such, data regarding the influence of the included risk factors and preliminary and exploratory. Moreover, DR grading was performed either through slit-lamp examinations or colour fundus photographs (focused on the optic disc and macula). Heterogeneity in imaging acquisition and quality may have reduced sensitivity, particularly for peripheral retinal lesions; the use of the 10-year follow-up mark may have led to an underestimation of the true lifetime prevalence of DR, as cases occurring and resolving before or after this interval would not be captured. Finally, the fact that this study was conducted at a single center in northern Portugal may limit the geographic generalizability of the findings. Differences in healthcare access, screening adherence, or socioeconomic factors in other regions may affect the generalization of these results.

## Conclusions

This retrospective study is a pioneering effort that provides an overview of the prevalence of DR in a cohort evaluated over 10 years. It is the only Portuguese study to date to estimate DR prevalence in this timeframe while also incorporating thorough baseline clinical/demographic data and risk factors. Despite the previously discussed limitations, the 10-year point prevalence of 6.12%, significantly lower than the available published evidence, warrants caution when citing the prevalence estimates derived from landmark 20th-century studies. This highlights the need to perform larger, multicentric studies to establish up‑to‑date, region‑specific figures and truly assess the burden of DR in the context of current Portuguese and international healthcare practices.

## References

[1] Nanayakkara N, Curtis AJ, Heritier S (2021). Impact of age at type 2 diabetes mellitus diagnosis on mortality and vascular complications: systematic review and meta-analyses. Diabetologia.

[2] Sarría-Santamera A, Orazumbekova B, Maulenkul T, Gaipov A, Atageldiyeva K (2020). The identification of diabetes mellitus subtypes applying cluster analysis techniques: a systematic review. Int J Environ Res Public Health.

[3] Magliano DJ, Boyko EJ (2021). IDF Diabetes Atlas. https://diabetesatlas.org/.

[4] Khalil H (2017). Diabetes microvascular complications-a clinical update. Diabetes Metab Syndr.

[5] Teo ZL, Tham YC, Yu M (2021). Global prevalence of diabetic retinopathy and projection of burden through 2045: systematic review and meta-analysis. Ophthalmology.

[6] Saúde DGd (2025). Diabetic retinopathy screening. https://normas.dgs.min-saude.pt/wp-content/uploads/2019/10/rastreio-da-retinopatia-diabetica.pdf.

[7] Fenwick E, Rees G, Pesudovs K, Dirani M, Kawasaki R, Wong TY, Lamoureux E (2012). Social and emotional impact of diabetic retinopathy: a review. Clin Exp Ophthalmol.

[8] Hertzberg SN, Jørstad ØK, Petrovski BÉ (2022). Transition from laser to intravitreal injections for diabetic retinopathy: hospital utilization and costs from an extended healthcare perspective. Int J Environ Res Public Health.

[9] Gardete-Correia L, Boavida JM, Raposo JF, Mesquita AC, Fona C, Carvalho R, Massano-Cardoso S (2010). First diabetes prevalence study in Portugal: PREVADIAB study. Diabet Med.

[10] Klein R, Klein BE, Moss SE, Davis MD, DeMets DL (1984). The Wisconsin Epidemiologic Study of Diabetic Retinopathy: III. Prevalence and risk of diabetic retinopathy when age at diagnosis is 30 or more years. Arch Ophthalmol.

[11] Stratton IM, Kohner EM, Aldington SJ, Turner RC, Holman RR, Manley SE, Matthews DR (2001). UKPDS 50: risk factors for incidence and progression of retinopathy in Type II diabetes over 6 years from diagnosis. Diabetologia.

[12] Broadbent DM, Scott JA, Vora JP, Harding SP (1999). Prevalence of diabetic eye disease in an inner city population: the Liverpool Diabetic Eye Study. Eye (Lond).

[13] Thomas RL, Dunstan F, Luzio SD (2012). Incidence of diabetic retinopathy in people with type 2 diabetes mellitus attending the Diabetic Retinopathy Screening Service for Wales: retrospective analysis. BMJ.

[14] Zhang X, Saaddine JB, Chou CF (2010). Prevalence of diabetic retinopathy in the United States, 2005-2008. JAMA.

[15] Dutra Medeiros M, Mesquita E, Papoila AL, Genro V, Raposo JF (2015). First diabetic retinopathy prevalence study in Portugal: RETINODIAB Study--evaluation of the screening programme for Lisbon and Tagus Valley region. Br J Ophthalmol.

[16] Dutra Medeiros M, Mesquita E, Gardete-Correia L (2015). First incidence and progression study for diabetic retinopathy in Portugal, the RETINODIAB Study: evaluation of the screening program for Lisbon Region. Ophthalmology.

[17] Jang HN, Moon MK, Koo BK (2022). Prevalence of diabetic retinopathy in undiagnosed diabetic patients: a nationwide population-based study. Diabetes Metab J.

[18] Zhao C, Wang W, Xu D, Li H, Li M, Wang F (2014). Insulin and risk of diabetic retinopathy in patients with type 2 diabetes mellitus: data from a meta-analysis of seven cohort studies. Diagn Pathol.

[19] Cai X, Chen Y, Yang W, Gao X, Han X, Ji L (2018). The association of smoking and risk of diabetic retinopathy in patients with type 1 and type 2 diabetes: a meta-analysis. Endocrine.

